# Supplementary material for: IGF-1 facilitates extinction of conditioned fear
Source: eLife. 2021 Apr 1;10:e67267. doi: 10.7554/eLife.67267 (PMC8043742; doi:10.7554/eLife.67267)
Supplement: Supplementary file 1. [file elife-67267-supp1.docx]

**Supplementary File 1_STATISTICS**

| FIGURE | STATISTIC | | CONDITION | | N | P VALUE | | | OUTPUT |
| --- | --- | --- | --- | --- | --- | --- | --- | --- | --- |
| **Figure 1: IGF-1 increases the excitability of IL-L5PNs** | | | | | | | | | |
| Fig. 1B | Paired t-test (two-tailed) | | _m_AHP (ACSF *vs* IGF-1) | | N=9 | 0.004 | | | ** |
|  |  |  | _m_AHP (NVP *vs* NVP+IGF-1) | | N=5 | 0.625 | | | Not significant |
|  | Unpaired t-test (two-tailed) | | _m_AHP (ACSF *vs* NVP) | | N=9 *vs* 5 | 0.529 | | | Not significant |
| Fig. 1C | Paired t-test (two-tailed) | | _s_AHP (ACSF *vs* IGF-1) | | N=9 | 0.004 | | | ** |
|  |  |  | _s_AHP (NVP *vs* NVP+IGF-1) | | N=5 | 0.625 | | | Not significant |
|  | Unpaired t-test (two-tailed) | | _s_AHP (ACSF *vs* NVP) | | N= 9 *vs* 5 | >0.999 | | | Not significant |
| Fig. 1E | Multiple t-test (post hoc multiple comparison Holm-Sildak method) | | 25 pA (ASCF *vs* IGF-1) | | N=10 | 0.410 | | | Not significant |
|  |  |  | 50 pA (ASCF *vs* IGF-1) | | N=10 | 0.273 | | | Not significant |
|  |  |  | 75 pA (ASCF *vs* IGF-1) | | N=10 | 0.533 | | | Not significant |
|  |  |  | 100 pA (ASCF *vs* IGF-1) | | N=10 | 0.01 | | | * |
|  |  |  | 125 pA (ASCF *vs* IGF-1) | | N=10 | 0.004 | | | ** |
|  |  |  | 150 pA (ASCF *vs* IGF-1) | | N=10 | 0.005 | | | ** |
|  |  |  | 175 pA (ASCF *vs* IGF-1) | | N=10 | 0.01 | | | * |
|  |  |  | 200 pA (ASCF *vs* IGF-1) | | N=10 | 0.005 | | | ** |
| **Figure 2: IGF-1 reduces _m_I_AHP_ and _s_I_AHP_ in IL neurons** | | | | | | | | | |
| Fig. 2B | Paired t-test (two-tailed) | | *_m_*I_AHP_ (ACSF *vs* IGF-1) | | N=9 | 0.004 | | | ****** |
|  |  |  | *_m_*I_AHP_ (NVP *vs* NVP+IGF-1) | | N=7 | 0.578 | | | Not significant |
|  | Unpaired t-test (two-tailed) | | *_m_*I_AHP_ (ACSF *vs* NVP) | | N=9 *vs* 7 | 0.918 | | | Not significant |
| Fig. 2C | Paired t-test (two-tailed) | | _m_I_AHP_ time curse (ACSF *vs* IGF-1) | | N=9 | <0.0001 | | | ******* |
|  |  |  | *_m_*I_AHP_ time curse (NVP *vs* NVP+IGF-1) | | N=7 | 0.140 | | | Not significant |
|  | Unpaired t-test (two-tailed) | | *_m_*I_AHP_ time curse (IGF-1 *vs* NVP+IGF-1) | | N=9 vs 7 | <0.0001 | | | *** |
| Fig. 2D | Paired t-test (two-tailed) | | _s_I_AHP_ (ACSF *vs* IGF-1) | | N=9 | 0.004 | | | ****** |
|  |  |  | *_s_*I_AHP_ (NVP *vs* NVP+IGF-1) | | N=7 | 0.163 | | | Not significant |
|  | Unpaired t-test (two-tailed) | | *_s_*I_AHP_ (ACSF *vs* NVP) | | N=9 vs 7 | 0.918 | | | Not significant |
| Fig. 2E | Paired t-test (two-tailed) | | _s_I_AHP_ time curse (ACSF vs IGF-1) | | N=9 | <0.0001 | | | *** |
|  |  |  | *_s_*I_AHP_ time curse (NVP *vs* NVP+IGF-1) | | N=7 | 0.301 | | | Not significant |
|  | Unpaired t-test (two-tailed) | | *_s_*I_AHP_ time curse (IGF-1 *vs* NVP+IGF-1) | | N=9 vs 7 | <0.0001 | | | *** |
| Fig. 2G | Paired t-test (two-tailed) | | *_m_*I_AHP_ GDPβs(ACSF *vs* IGF-1) | | N=8 | 0.109 | | | Not significant |
|  |  |  | _s_I_AHP_ GDPβs (ACSF *vs* IGF-1) | | N=8 | 0.023 | | | * |
| **Figure 3:** **IGF-1 induces long-term potentiation in IL-PNL5** | | | | | | | | | |
| Fig. 3A | Paired t-test (two-tailed) | | EPSC (ACSF *vs* IGF-1) | | N=5 | 0.0001 | | | *** |
|  |  |  | EPSC (NVP *vs* NVP+IGF-1) | | N=5 | 0.244 | | | Not significant |
|  | Unpaired t-test (two-tailed) | | EPSC (IGF-1 *vs* NVP+IGF-1) | | N=5 *vs* 5 | 0.002 | | | *** |
| Fig. 3B | Paired t-test (two-tailed) | | PPR_EPSC_ (ACSF *vs* IGF-1) | | N=5 | 0.004 | | | ** |
| Fig. 3D | Paired t-test (two-tailed) | | EPSC no stim (ACSF *vs* IGF-1) | | N=6 | <0.0001 | | | *** |
|  |  |  | PPR_EPSC no stim_ (ACSF *vs* IGF-1) | | N=6 | 0.645 | | | Not significant |
| Fig. 3A-D | Unpaired t-test (two-tailed) | | EPSC (IGF-1 *vs* IGF-1(no stim)) | | N=5 v*s* 6 | 0.002 | | | ** |
| Fig. 3E | Paired t-test (two-tailed) | | IPSC (ACSF *vs* IGF-1) | | N=6 | <0.001 | | | *** |
|  |  |  | IPSC (NVP *vs* NVP+IGF-1) | | N=6 | 0.220 | | | Not significant |
|  | Unpaired t-test (two-tailed) | | IPSC (IGF-1 *vs* NVP+IGF-1) | | N=6 *vs* 6 | 0.008 | | | ** |
| Fig. 3F | Paired t-test (two-tailed) | | PPR_IPSC_ (ACSF *vs* IGF-1) | | N=6 | 0.044 | | | * |
| Fig. 3H | Paired t-test (two-tailed) | | IPSC (ACSF vs IGF-1) | | N=6 | 0.0006 | | | *** |
|  |  |  | PPR_IPSC no stim_ (ACSF *vs* IGF-1) | | N=6 | 0.327 | | | Not significant |
| Fig. 3E-H | Unpaired t-test (two-tailed) | | IPSC (IGF-1 *vs* IGF-1(no stim)) | | N=6 v*s* 5 | 0.004 | | | ** |
| Fig. 3I | Test for normal distribution Kolmogorov-Smirnov test  Paired t-test (two-tailed) | | % PSP (ACSF *vs* IGF-1) | | N=6 | <0.000002 | | | *** |
| Fig. 3K |  |  | PSP amplitude (ACSF *vs* IGF-1) | | N=6 | 0.016 | | | * |
|  |  |  | PSP area (ACSF *vs* IGF-1) | | N=6 | 0.052 | | | Not significant |
|  |  |  | PSP Tau ON (ACSF *vs* IGF-1) | | N=6 | 0.349 | | | Not significant |
|  |  |  | PSP Tau OFF (ACSF *vs* IGF-1) | | N=6 | 0.108 | | | Not significant |
| Fig. 3M | One-way ANOVA  (post hoc Dunnett’s multiple comparisons test) | | AP (ACSF *vs* IGF-1) 5 min | | N=6 | 0.640 | | | Not significant |
|  |  |  | AP (ACSF *vs* IGF-1) 10 min | | N=6 | 0.315 | | | Not significant |
|  |  |  | AP (ACSF *vs* IGF-1) 15 min | | N=6 | 0.005 | | | ** |
| Fig. 3N | Test for normal distribution Kolmogorov-Smirnov test  Paired t-test (two-tailed) | | AP amplitude (ACSF *vs* IGF-1) | | N=6 | 0.031 | | | * |
|  |  |  | AP half-width (ACSF *vs* IGF-1) | | N=6 | 0.100 | | | Not significant |
|  |  |  | AP time to rise slope (ACSF *vs* IGF-1) | | N=6 | 0.451 | | | Not significant |
|  |  |  | AP rise slope (ACSF *vs* IGF-1) | | N=6 | 0.843 | | | Not significant |
|  |  |  | AP time to decay slope (ACSF *vs* IGF-1) | | N=6 | 0.666 | | | Not significant |
|  |  |  | AP decay slope (ACSF *vs* IGF-1) | | N=6 | 0.133 | | | Not significant |
| **Figure 4:** **IGF-1 facilitates the memory of extinction by reducing mIAHP and sIAHP, increasing the firing frequency** | | | | | | | | | |
| Fig.4B | One-way ANOVA  (post hoc Turkey’s multiple comparisons test) | | **Cond** Session 1 (Saline *vs* IGF-1) | | N=11-14 | 0.141 | | | Not significant |
|  |  |  | **Cond** Session 1 (IGF-1 *vs* NVP+IGF-1) | | N=14-12 | 0.984 | | | Not significant |
|  |  |  | **Cond** Session 1 (Saline *vs* NVP+IGF-1) | | N=11-12 | 0.118 | | | Not significant |
|  |  |  | **Cond** Session 2 (Saline *vs* IGF-1) | | N=11-14 | 0.293 | | | Not significant |
|  |  |  | **Cond** Session 2 (IGF-1 *vs* NVP+IGF-1) | | N=14-12 | 0.911 | | | Not significant |
|  |  |  | **Cond** Session 2 (Saline *vs* NVP+IGF-1) | | N=11-12 | 0.166 | | | Not significant |
|  |  |  | **Cond** Session 3 (Saline *vs* IGF-1) | | N=11-14 | 0.332 | | | Not significant |
|  |  |  | **Cond** Session 3 (IGF-1 *vs* NVP+IGF-1) | | N=14-12 | 0.977 | | | Not significant |
|  |  |  | **Cond** Session 3 (Saline *vs* NVP+IGF-1) | | N=11-12 | 0.459 | | | Not significant |
| Fig.4B | One-way ANOVA  (post hoc Turkey’s multiple comparisons test) | | **Ext** Session 1 (Saline *vs* IGF-1) | | N=11-14 | 0.984 | | | Not significant |
|  |  |  | **Ext** Session 1 (IGF-1 *vs* NVP+IGF-1) | | N=14-12 | 0.719 | | | Not significant |
|  |  |  | **Ext** 3 Session 1 (Saline *vs* NVP+IGF-1) | | N=11-12 | 0.840 | | | Not significant |
|  |  |  | **Ext** Session 2 (Saline *vs* IGF-1) | | N=11-14 | 0.484 | | | Not significant |
|  |  |  | **Ext** Session 2 (IGF-1 *vs* NVP+IGF-1) | | N=14-12 | 0.528 | | | Not significant |
|  |  |  | **Ext** Session 2 (Saline *vs* NVP+IGF-1) | | N=11-12 | 0.992 | | | Not significant |
|  |  |  | **Ext** Session 3 (Saline *vs* IGF-1) | | N=11-14 | 0.688 | | | Not significant |
|  |  |  | **Ext** Session 3 (IGF-1 *vs* NVP+IGF-1) | | N=14-12 | >0.999 | | | Not significant |
|  |  |  | **Ext** Session 3 (Saline *vs* NVP+IGF-1) | | N=11-12 | 0.680 | | | Not significant |
|  |  |  | **Ext** Session 4 (Saline *vs* IGF-1) | | N=11-14 | 0.892 | | | Not significant |
|  |  |  | **Ext** Session 4 (IGF-1 *vs* NVP+IGF-1) | | N=14-12 | 0.534 | | | Not significant |
|  |  |  | **Ext** Session 4 (Saline *vs* NVP+IGF-1) | | N=11-12 | 0.844 | | | Not significant |
|  |  |  | **Ext** Session 5 (Saline *vs* IGF-1) | | N=11-14 | 0.927 | | | Not significant |
|  |  |  | **Ext** Session 5 (IGF-1 *vs* NVP+IGF-1) | | N=14-12 | 0.543 | | | Not significant |
|  |  |  | **Ext** Session 5 (Saline *vs* NVP+IGF-1) | | N=11-12 | 0.369 | | | Not significant |
|  |  |  | **Ext** Session 6 (Saline *vs* IGF-1) | | N=11-14 | 0.504 | | | Not significant |
|  |  |  | **Ext** Session 6 (IGF-1 *vs* NVP+IGF-1) | | N=14-12 | 0.170 | | | Not significant |
|  |  |  | **Ext** Session 6 (Saline *vs* NVP+IGF-1) | | N=11-12 | 0.820 | | | Not significant |
|  |  |  | **Ext** Session 7 (Saline *vs* IGF-1) | | N=11-14 | 0.978 | | | Not significant |
|  |  |  | **Ext** Session 7 (IGF-1 *vs* NVP+IGF-1) | | N=14-12 | 0.493 | | | Not significant |
|  |  |  | **Ext** Session 7 (Saline *vs* NVP+IGF-1) | | N=11-12 | 0.418 | | | Not significant |
|  |  |  | **Ext** Session 8 (Saline *vs* IGF-1) | | N=11-14 | 0.650 | | | Not significant |
|  |  |  | **Ext** Session 8 (IGF-1 *vs* NVP+IGF-1) | | N=14-12 | 0.302 | | | Not significant |
|  |  |  | **Ext** Session 8 (Saline *vs* NVP+IGF-1) | | N=11-12 | 0.075 | | | Not significant |
|  |  |  | **Ext** Session 9 (Saline *vs* IGF-1) | | N=11-14 | 0.699 | | | Not significant |
|  |  |  | **Ext** Session 9 (IGF-1 *vs* NVP+IGF-1) | | N=14-12 | 0.111 | | | Not significant |
|  |  |  | **Ext** Session 9 (Saline *vs* NVP+IGF-1) | | N=11-12 | 0.502 | | | Not significant |
|  |  |  | **Ext** Session 10 (Saline *vs* IGF-1) | | N=11-14 | 0.429 | | | Not significant |
|  |  |  | **Ext** Session 10 (IGF-1 *vs* NVP+IGF-1) | | N=14-12 | 0.065 | | | Not significant |
|  |  |  | **Ext** Session 10 (Saline *vs* NVP+IGF-1) | | N=11-12 | 0.629 | | | Not significant |
|  |  |  | **Ext** Session 11 (Saline *vs* IGF-1) | | N=11-14 | 0.924 | | | Not significant |
|  |  |  | **Ext** Session 11 (IGF-1 *vs* NVP+IGF-1) | | N=14-12 | 0.663 | | | Not significant |
|  |  |  | **Ext** Session 11 (Saline *vs* NVP+IGF-1) | | N=11-12 | 0.901 | | | Not significant |
|  |  |  | **Ext** Session 12 (Saline *vs* IGF-1) | | N=11-14 | 0.368 | | | Not significant |
|  |  |  | **Ext** Session 12 (IGF-1 *vs* NVP+IGF-1) | | N=14-12 | 0.083 | | | Not significant |
|  |  |  | **Ext** Session 12 (Saline *vs* NVP+IGF-1) | | N=11-12 | 0.761 | | | Not significant |
|  |  |  | **Ext** Session 13 (Saline *vs* IGF-1) | | N=11-14 | 0.356 | | | Not significant |
|  |  |  | **Ext** Session 13 (IGF-1 *vs* NVP+IGF-1) | | N=14-12 | 0.138 | | | Not significant |
|  |  |  | **Ext** Session 13 (Saline *vs* NVP+IGF-1) | | N=11-12 | 0.900 | | | Not significant |
|  |  |  | **Ext** Session 14 (Saline *vs* IGF-1) | | N=11-14 | 0.030 | | | * |
|  |  |  | **Ext** Session 14 (IGF-1 *vs* NVP+IGF-1) | | N=14-12 | 0.085 | | | Not significant |
|  |  |  | **Ext** Session 14 (Saline *vs* NVP+IGF-1) | | N=11-12 | 0.816 | | | Not significant |
|  |  |  | **Ext** Session 15 (Saline *vs* IGF-1) | | N=11-14 | 0.099 | | | Not significant |
|  |  |  | **Ext** Session 15 (IGF-1 *vs* NVP+IGF-1) | | N=14-12 | 0.021 | | | # |
|  |  |  | **Ext** Session 15 (Saline *vs* NVP+IGF-1) | | N=11-12 | 0.862 | | | Not significant |
|  |  |  | **Ext** Session 16 (Saline *vs* IGF-1) | | N=11-14 | 0.428 | | | Not significant |
|  |  |  | **Ext** Session 16 (IGF-1 *vs* NVP+IGF-1) | | N=14-12 | 0.059 | | | Not significant |
|  |  |  | **Ext** Session 16 (Saline *vs* NVP+IGF-1) | | N=11-12 | 0.561 | | | Not significant |
|  |  |  | **Ext** Session 17 (Saline *vs* IGF-1) | | N=11-14 | 0.192 | | | Not significant |
|  |  |  | **Ext** Session 17 (IGF-1 *vs* NVP+IGF-1) | | N=14-12 | 0.141 | | | Not significant |
|  |  |  | **Ext** Session 17 (Saline *vs* NVP+IGF-1) | | N=11-12 | 0.999 | | | Not significant |
|  |  |  | **Ext** Session 18 (Saline *vs* IGF-1) | | N=11-14 | 0.277 | | | Not significant |
|  |  |  | **Ext** Session 18 (IGF-1 *vs* NVP+IGF-1) | | N=14-12 | 0.576 | | | Not significant |
|  |  |  | **Ext** Session 18 (Saline *vs* NVP+IGF-1) | | N=11-12 | 0.816 | | | Not significant |
|  |  |  | **Ext** Session 19 (Saline *vs* IGF-1) | | N=11-14 | 0.253 | | | Not significant |
|  |  |  | **Ext** Session 19 (IGF-1 *vs* NVP+IGF-1) | | N=14-12 | 0.007 | | | ## |
|  |  |  | **Ext** Session 19 (Saline *vs* NVP+IGF-1) | | N=11-12 | 0.340 | | | Not significant |
|  |  |  | **Ext** Session 20 (Saline *vs* IGF-1) | | N=11-14 | 0.578 | | | Not significant |
|  |  |  | **Ext** Session 20 (IGF-1 *vs* NVP+IGF-1) | | N=14-12 | 0.0002 | | | ### |
|  |  |  | **Ext** Session 20 (Saline *vs* NVP+IGF-1) | | N=11-12 | 0.008 | | | ** |
| Fig.4B | One-way ANOVA  (post hoc Turkey’s multiple comparisons test) | | **Test** Session 1 (Saline *vs* IGF-1) | | N=11-14 | 0.117 | | | Not significant |
|  |  |  | **Test** Session 1 (IGF-1 *vs* NVP+IGF-1) | | N=14-12 | 0.060 | | | Not significant |
|  |  |  | **Test** Session 1 (Saline *vs* NVP+IGF-1) | | N=11-12 | 0.973 | | | Not significant |
|  |  |  | **Test** Session 2 (Saline *vs* IGF-1) | | N=11-14 | 0.423 | | | Not significant |
|  |  |  | **Test** Session 2 (IGF-1 *vs* NVP+IGF-1) | | N=14-12 | 0.153 | | | Not significant |
|  |  |  | **Test** Session 2 (Saline *vs* NVP+IGF-1) | | N=11-12 | 0.849 | | | Not significant |
|  |  |  | **Test** Session 3 (Saline *vs* IGF-1) | | N=11-14 | 0.204 | | | Not significant |
|  |  |  | **Test** Session 3 (IGF-1 *vs* NVP+IGF-1) | | N=14-12 | 0.012 | | | # |
|  |  |  | **Test** Session 3 (Saline *vs* NVP+IGF-1) | | N=11-12 | 0.470 | | | Not significant |
|  |  |  | **Test** Session 4 (Saline *vs* IGF-1) | | N=11-14 | 0.043 | | | * |
|  |  |  | **Test** Session 4 (IGF-1 *vs* NVP+IGF-1) | | N=14-12 | 0.002 | | | ## |
|  |  |  | **Test** Session 4 (Saline *vs* NVP+IGF-1) | | N=11-12 | 0.486 | | | Not significant |
|  |  |  | **Test** Session 5 (Saline *vs* IGF-1) | | N=11-14 | 0.008 | | | ** |
|  |  |  | **Test** Session 5 (IGF-1 *vs* NVP+IGF-1) | | N=14-12 | 0.0003 | | | ### |
|  |  |  | **Test** Session 5 (Saline *vs* NVP+IGF-1) | | N=11-12 | 0.850 | | | Not significant |
| Fig.4D | One-way ANOVA  (post hoc Turkey’s multiple comparisons test) | | _m_I_AHP_ (Ext-Saline *vs* Ext-IGF-1) | | N=11-7 | 0.049 | | | * |
|  |  |  | _m_I_AHP_ (Ext-IGF-1 *vs* Ext-NVP+IGF-1) | | N=7-9 | 0.01 | | | ** |
|  |  |  | _m_I_AHP_ (Ext-Saline *vs* Ext-NVP+IGF-1) | | N=11-9 | 0.663 | | | Not significant |
| Fig.4E | One-way ANOVA  (post hoc Turkey’s multiple comparisons test) | | _s_I_AHP_ (Ext-Saline *vs* Ext-IGF-1) | | N=11-7 | 0.049 | | | * |
|  |  |  | _s_I_AHP_ (Ext-IGF-1 *vs* Ext-NVP+IGF-1) | | N=7-9 | 0.0006 | | | *** |
|  |  |  | _s_I_AHP_ (Ext-Saline *vs* Ext-NVP+IGF-1) | | N=11-9 | 0.096 | | | Not significant |
| Fig.4F |  | | Correlation between (_m_I_AHP_-%freezing) | | N=10 | r=0.763 | | | - |
|  |  |  | Correlation between (_s_I_AHP_-%freezing) | | N=10 | r=787 | | | - |
| Fig.4G | Two-way ANOVA  post hoc Turkey’s multiple comparisons test) | | **25 pA** (Ext-IGF-1 *vs* Ext-NVP+IGF-1) | | N=7-6 | 0.991 | | | Not significant |
|  |  |  | **25 pA** (Ext-IGF-1 *vs* Ext-Saline) | | N=7-10 | 0.989 | | | Not significant |
|  |  |  | **25 pA** (Ext-NVP+IGF-1 *vs* Ext-Saline) | | N=6-10 | >0.999 | | | Not significant |
|  |  |  | **50 pA** (Ext-IGF-1 *vs* Ext-NVP+IGF-1) | | N=7-6 | 0.903 | | | Not significant |
|  |  |  | **50 pA** (Ext-IGF-1 *vs* Ext-Saline) | | N=7-10 | 0.937 | | | Not significant |
|  |  |  | **50 pA** (Ext-NVP+IGF-1 *vs* Ext-Saline) | | N=6-10 | 0.990 | | | Not significant |
|  |  |  | **75 pA** (Ext-IGF-1 *vs* Ext-NVP+IGF-1) | | N=7-6 | 0.897 | | | Not significant |
|  |  |  | **75 pA** (Ext-IGF-1 *vs* Ext-Saline) | | N=7-10 | 0.876 | | | Not significant |
|  |  |  | **75 pA** (Ext-NVP+IGF-1 *vs* Ext-Saline) | | N=6-10 | >0.999 | | | Not significant |
|  |  |  | **100 pA** (Ext-IGF-1 *vs* Ext-NVP+IGF-1) | | N=7-6 | 0.655 | | | Not significant |
|  |  |  | **100 pA** (Ext-IGF-1 *vs* Ext-Saline) | | N=7-10 | 0.778 | | | Not significant |
|  |  |  | **100 pA** (Ext-NVP+IGF-1 *vs* Ext-Saline) | | N=6-10 | 0.951 | | | Not significant |
|  |  |  | **125 pA** (Ext-IGF-1 *vs* Ext-NVP+IGF-1) | | N=7-6 | 0.611 | | | Not significant |
|  |  |  | **125 pA** (Ext-IGF-1 *vs* Ext-Saline) | | N=7-10 | 0.826 | | | Not significant |
|  |  |  | **125 pA** (Ext-NVP+IGF-1 *vs* Ext-Saline) | | N=6-10 | 0.890 | | | Not significant |
|  |  |  | **150 pA** (Ext-IGF-1 *vs* Ext-NVP+IGF-1) | | N=7-6 | 0.339 | | | Not significant |
|  |  |  | **150 pA** (Ext-IGF-1 *vs* Ext-Saline) | | N=7-10 | 0.863 | | | Not significant |
|  |  |  | **150 pA** (Ext-NVP+IGF-1 *vs* Ext-Saline) | | N=6-10 | 0.563 | | | Not significant |
|  |  |  | **175 pA** (Ext-IGF-1 *vs* Ext-NVP+IGF-1) | | N=7-6 | 0.101 | | | Not significant |
|  |  |  | **175 pA** (Ext-IGF-1 *vs* Ext-Saline) | | N=7-10 | 0.397 | | | Not significant |
|  |  |  | **175 pA** (Ext-NVP+IGF-1 *vs* Ext-Saline) | | N=6-10 | 0.514 | | | Not significant |
|  |  |  | **200 pA** (Ext-IGF-1 *vs* Ext-NVP+IGF-1) | | N=7-6 | 0.046 | | | # |
|  |  |  | **200 pA** (Ext-IGF-1 *vs* Ext-Saline) | | N=7-10 | 0.265 | | | Not significant |
|  |  |  | **200 pA** (Ext-NVP+IGF-1 *vs* Ext-Saline) | | N=6-10 | 0.435 | | | Not significant |
|  |  |  | **225 pA** (Ext-IGF-1 *vs* Ext-NVP+IGF-1) | | N=7-6 | 0.049 | | | # |
|  |  |  | **225 pA** (Ext-IGF-1 *vs* Ext-Saline) | | N=7-10 | 0.265 | | | Not significant |
|  |  |  | **225 pA** (Ext-NVP+IGF-1 *vs* Ext-Saline) | | N=6-10 | 0.435 | | | Not significant |
|  |  |  | **250 pA** (Ext-IGF-1 *vs* Ext-NVP+IGF-1) | | N=7-6 | 0.013 | | | # |
|  |  |  | **250 pA** (Ext-IGF-1 *vs* Ext-Saline) | | N=7-10 | 0.016 | | | * |
|  |  |  | **250 pA** (Ext-NVP+IGF-1 *vs* Ext-Saline) | | N=6-10 | 0.724 | | | Not significant |
|  |  |  | **275 pA** (Ext-IGF-1 *vs* Ext-NVP+IGF-1) | | N=7-6 | 0.007 | | | ## |
|  |  |  | **275 pA** (Ext-IGF-1 *vs* Ext-Saline) | | N=7-10 | 0.017 | | | * |
|  |  |  | **275 pA** (Ext-NVP+IGF-1 *vs* Ext-Saline) | | N=6-10 | 0.724 | | | Not significant |
|  |  |  | **300 pA** (Ext-IGF-1 *vs* Ext-NVP+IGF-1) | | N=7-6 | 0.0003 | | | ### |
|  |  |  | **300 pA** (Ext-IGF-1 *vs* Ext-Saline) | | N=7-10 | 0.0001 | | | *** |
|  |  |  | **300 pA** (Ext-NVP+IGF-1 *vs* Ext-Saline) | | N=6-10 | 0.903 | | | Not significant |
| **Figure 5: IGF-1 decreases the frequency of mEPSC** | | | | | | | | | |
| Fig. 5B | One-way ANOVA  (post hoc Turkey’s multiple comparisons test) | | mEPSC (Ext-Saline *vs* Ext-IGF-1) | | N=5-6 | 0.017 | | | * |
|  |  |  | mEPSC (Ext-Saline *vs* Ext-NVP+IGF-1) | | N=5-6 | 0.973 | | | Not significant |
|  |  |  | mEPSC (Ext-IGF-1 *vs* Ext-NVP+IGF-1) | | N=6-6 | 0.020 | | | * |
| Fig. 5C | Kolmogorov-Smirnov test | | mEPSC (Ext-Saline *vs* Ext-IGF-1) | | - | <0.0001 | | | *** |
|  |  |  | mEPSC (Ext-Saline *vs* Ext-NVP+IGF-1) | | - | 0.003 | | | ** |
|  |  |  | mEPSC (Ext-IGF-1 *vs* Ext-NVP+IGF-1) | | - | <0.0001 | | | ### |
| Fig. 5D | One-way ANOVA  (post hoc Turkey’s multiple comparisons test) | | mEPSC (Ext-Saline *vs* Ext-IGF-1) | | N=6 | 0.781 | | | Not significant |
|  |  |  | mEPSC (Ext-Saline vs Ext-NVP+IGF-1) | | N=5-6 | 0.972 | | | Not significant |
|  |  |  | mEPSC (Ext-IGF-1 vs Ext-NVP+IGF-1) | | N=6-6 | 0.885 | | | Not significant |
| Fig.5E | Kolmogorov-Smirnov test | | mEPSC (Ext-Saline vs Ext-IGF-1) | | - | 0.015 | | | * |
|  |  |  | mEPSC (Ext-Saline vs Ext-NVP+IGF-1) | | - | <0.0001 | | | *** |
|  |  |  | mEPSC (Ext-IGF-1 vs Ext-NVP+IGF-1) | | - | <0.0001 | | | ### |
| **Supplementary Figure 1: IGF-1 has no effect on fAHP and fIAHP** | | | | | | | | | |
| Fig. S1B | Paired t-test (two-tailed) | | _f_AHP (ACSF *vs* IGF-1) | | N=7 | 0.687 | | | Not significant |
|  |  |  | _f_AHP (NVP *vs* NVP+IGF-1) | | N=5 | 0.062 | | | Not significant |
|  | Unpaired t-test (two-tailed) | | _f_AHP (ACSF *vs* NVP) | | N=7-5 | 0.106 | | | Not significant |
| Fig. S1D | Paired t-test (two-tailed) | | *_m_*I_AHP_ (ACSF *vs* IGF-1) | | N=7 | 0.250 | | | Not significant |
|  |  |  | *_m_*I_AHP_ (NVP *vs* NVP+IGF-1) | | N=6 | 0.687 | | | Not significant |
| **Supplementary Figure 2: Action potential properties and resting membrane of IL-L5PNs** | | | | | | | | | |
| Fig. S2A | | Test for normal distribution Kolmogorov-Smirnov test  Paired t-test (two-tailed) or Wilcoxon test | | AP amplitude (ACSF *vs* IGF-1) | N=9 | | 0.286 | Not significant | |
| Fig. S2B | |  |  | AP threshold (ACSF *vs* IGF-1) | N=9 | | 0.781 | Not significant | |
| Fig. S2C | |  |  | AP half-width (ACSF *vs* IGF-1) | N=9 | | 0.734 | Not significant | |
| Fig. S2D | |  |  | AP time to rise slope (ACSF *vs* IGF-1) | N=9 | | 0.478 | Not significant | |
| Fig. S2E | |  |  | AP max rise slope (ACSF *vs* IGF-1) | N=9 | | 0.190 | Not significant | |
| Fig. S2F | |  |  | AP time to decay slope (ACSF *vs* IGF-1) | N=9 | | 0.912 | Not significant | |
| Fig. S2G | |  |  | AP max decay slope (ACSF *vs* IGF-1) | N=9 | | 0.912 | Not significant | |
| Fig. S2H | |  |  | Rm (ACSF *vs* IGF-1) | N=12 | | <0.0005 | *** | |
| **Supplementary Figure 3: Time curse of mIAHP and sIAHP** | | | | | | | | | |
| Fig. S3A | Paired t-test (two-tailed) | | _m_I_AHP_ protocol | | N=4 | | 0.320 | Not significant | |
|  |  |  | _s_I_AHP_ protocol | | N=4 | | 0.672 | Not significant | |
| Fig. S3B | Paired t-test (two-tailed) | | _m_I_AHP_ time curse (ACSF *vs* washing IGF-1) | | N=7 | | 0.006 | ** | |
|  |  |  | _m_I_AHP_ time curse (ACSF *vs* washing IGF-1) | | N=5 | | 0.0008 | *** | |
|  | Unpaired t-test (two-tailed) | | _m_I_AHP_ LTD | | N=7-5 | | 0.882 | Not significant | |
| Fig. S3C | Paired t-test (two-tailed) | | _s_I_AHP_ time curse (ACSF *vs* washing IGF-1) | | N=7 | | 0.002 | ** | |
|  | Paired t-test (two-tailed) | | _s_I_AHP_ time curse (ACSF *vs* washing IGF-1) | | N=5 | | 0.003 | ** | |
|  | Unpaired t-test (two-tailed) | | _s_I_AHP_ LTD | | N=7-5 | | 0.838 | Not significant | |
| **Supplementary Figure 4: Protocol of stimulation does not induce plasticity** | | | | | | | | | |
| Fig. S4A | Paired t-test (two-tailed) | | % PSP (ACSF before *vs* after stim.) | | N=6 | | 0.437 | Not significant | |
| Fig. S4B | One-way ANOVA  (post hoc Dunnett’s multiple comparisons test) | | AP (ACSF) 5 min | | N=6 | | 0.443 | Not significant | |
|  |  |  | AP (ACSF) 10 min | | N=6 | | 0.943 | Not significant | |
|  |  |  | AP (ACSF) 15 min | | N=6 | | 0.988 | Not significant | |
| **Supplementary Figure 5: The application of IGF-1 for 10 min is sufficient to induce plasticity** | | | | | | | | | |
| Fig. S5A | Paired t-test (two-tailed) | | % PSP (ACSF *vs* washing IGF-1) | | N=4 | | 0.0010 | *** | |
| Fig. S5B | One-way ANOVA  (post hoc Dunnett’s multiple comparisons test) | | AP (ACSF vs IGF-1) 5 min | | N=4 | | 0.068 | Not significant | |
|  |  |  | AP (ACSF vs IGF-1) 10 min | | N=4 | | 0.013 | * | |
| **Supplementary Figure 6:** **Protocol of behavior** | | | | | | | | | |
| Fig.S6B | One-way ANOVA  (post hoc Turkey’s multiple comparisons test) | | **Cond** Session 1 (Cond *vs* Ext) | | N=7-11 | | 0.847 | Not significant | |
|  |  |  | **Cond** Session 2 (Cond *vs* Ext) | | N=7-11 | | >0.999 | Not significant | |
|  |  |  | **Cond** Session 3 (Cond *vs* Ext) | | N=7-11 | | 0.999 | Not significant | |
| Fig. S6B | One-way ANOVA  (post hoc Turkey’s multiple comparisons test) | | **Test** Session 1 (Cond *vs* Ext) | | N=7-11 | | 0.361 | Not significant | |
|  |  |  | **Test** Session 2 (Cond *vs* Ext) | | N=7-11 | | 0.040 | ***** | |
|  |  |  | **Test** Session 3 (Cond *vs* Ext) | | N=7-11 | | 0.0041 | ****** | |
|  |  |  | **Test** Session 4 (Cond *vs* Ext) | | N=7-11 | | 0.0003 | ******* | |
|  |  |  | **Test** Session 5 (Cond *vs* Ext) | | N=7-11 | | 0.0006 | ******* | |
| **Supplementary Figure 7: Hyperpolarized potentials recording after behaviors** | | | | | | | | | |
| Fig. S7A | | One-way ANOVA  (post hoc Turkey’s multiple comparisons test) | | *_f_*AHP (NAÏVE *vs* EXT) | N=10-6 | | 0.006 | ** | |
|  |  |  |  | *_f_*AHP (NAÏVE *vs* Ext-Saline) | N=10-10 | | 0.011 | * | |
|  |  |  |  | *_f_*AHP (NAÏVE *vs* Ext-IGF-1) | N=10-7 | | 0.014 | * | |
|  |  |  |  | *_f_*AHP (NAÏVE *vs* EXT-NVP+IGF-1) | N=10-8 | | 0.525 | Not significant | |
|  |  |  |  | *_f_*AHP (EXT *vs* Ext-Saline) | N=6-10 | | 0.958 | Not significant | |
|  |  |  |  | *_f_*AHP (EXT *vs* Ext-IGF-1) | N=6-7 | | 0.992 | Not significant | |
|  |  |  |  | *_f_*AHP (EXT *vs* Ext-NVP+IGF-1) | N=6-8 | | 0.227 | Not significant | |
|  |  |  |  | *_f_*AHP (Ext-Saline *vs* Ext-IGF-1) | N=10-7 | | 0.999 | Not significant | |
|  |  |  |  | *_f_*AHP (Ext-Saline *vs* Ext-NVP+IGF-1) | N=10-8 | | 0.456 | Not significant | |
|  |  |  |  | *_f_*AHP (Ext-IGF-1 *vs* Ext-NVP+IGF-1) | N=7-8 | | 0.408 | Not significant | |
| Fig. S7B | | One-way ANOVA  (post hoc Turkey’s multiple comparisons test) | | *_m_*AHP (NAÏVE *vs* EXT) | N=16-6 | | 0.029 | * | |
|  |  |  |  | *_m_*AHP (NAÏVE *vs* Ext-Saline) | N=16-13 | | 0.028 | * | |
|  |  |  |  | *_m_*AHP (NAÏVE *vs* Ext-IGF-1) | N=16-6 | | 0.007 | ** | |
|  |  |  |  | *_m_*AHP (NAÏVE *vs* Ext-NVP+IGF-1) | N=16-11 | | 0.033 | * | |
|  |  |  |  | *_m_*AHP (EXT *vs* Ext-Saline) | N=6-13 | | 0.967 | Not significant | |
|  |  |  |  | *_m_*AHP (EXT *vs* Ext-IGF-1) | N=6-6 | | 0.993 | Not significant | |
|  |  |  |  | *_m_*AHP (EXT *vs* Ext-NVP+IGF-1) | N=6-11 | | 0.979 | Not significant | |
|  |  |  |  | *_m_*AHP (Ext-Saline *vs* Ext-IGF-1) | N=13-6 | | 0.786 | Not significant | |
|  |  |  |  | *_m_*AHP (Ext-Saline *vs* Ext-NVP+IGF-1) | N=13-11 | | >0.999 | Not significant | |
|  |  |  |  | *_m_*AHP (Ext-IGF-1 *vs* Ext-NVP+IGF-1) | N=6-11 | | 0.833 | Not significant | |
| Fig. S7C | | One-way ANOVA  (post hoc Turkey’s multiple comparisons test) | | *_s_*AHP (NAÏVE *vs* EXT) | N=12-6 | | 0.313 | Not significant | |
|  |  |  |  | *_s_*AHP (NAÏVE *vs* Ext-Saline) | N=12-11 | | 0.063 | Not significant | |
|  |  |  |  | *_s_*AHP (NAÏVE *vs* Ext-IGF-1) | N=12-6 | | 0.012 | * | |
|  |  |  |  | *_s_*AHP (NAÏVE *vs* Ext-NVP+IGF-1) | N=12-11 | | 0.074 | Not significant | |
|  |  |  |  | *_s_*AHP (EXT *vs* Ext-Saline) | N=6-11 | | 0.996 | Not significant | |
|  |  |  |  | *_s_*AHP (EXT *vs* Ext-IGF-1) | N=6-6 | | 0.707 | Not significant | |
|  |  |  |  | *_s_*AHP (EXT *vs* Ext-NVP+IGF-1) | N=6-11 | | 0.998 | Not significant | |
|  |  |  |  | *_s_*AHP (Ext-Saline *vs* Ext-IGF-1) | N=11-6 | | 0.807 | Not significant | |
|  |  |  |  | *_s_*AHP (Ext-Saline *vs* Ext-NVP+IGF-1) | N=11-11 | | >0.999 | Not significant | |
|  |  |  |  | *_s_*AHP (Ext-IGF-1 *vs* Ext-NVP+IGF-1) | N=6-11 | | 0.777 | Not significant | |
